# Intelligent Interface Detection of Frozen Rock Masses Using Measurement While Drilling Data and Change-Point Analysis

**DOI:** 10.3390/s26082397

**Published:** 2026-04-14

**Authors:** Fei Gao, Hui Chen, Xiujun Wu, Huijie Zhai, Yuanxiang Mu

**Affiliations:** 1 School of Geology and Mining Engineering, Xinjiang University, Urumqi 830047, China; 107556525523@stu.xju.edu.cn (F.G.);; 2 Key Laboratory of Green and Efficient Mining and Ecological Restoration in High-Altitude Arid Regions of Xinjiang, Urumqi 830047, China; 3 Xinjiang Green Blasting Engineering Technology Research Center, Urumqi 830047, China

**Keywords:** Measurement While Drilling (MWD), rock layer interface recognition, temperature variation conditions, change-point detection

## Abstract

**Highlights:**

**What are the main findings?**

- Sub-zero temperatures (−20 °C) intensify ice–rock coupling, causing severe high-frequency volatility and non-linear baseline shifts in Measurement While Drilling (MWD) sensor signals, specifically increasing torque and feed pressure while decreasing rotational speed.
- A proposed dual-mechanism change-point detection algorithm, integrated with Z-score normalization, successfully filters out temperature-induced “pseudo-interfaces,” achieving a rock layer interface prediction error of less than 1.5 mm.

**What are the implications of the main findings?**

- The study provides a robust signal-processing framework that effectively compensates for extreme-temperature data drift, significantly enhancing the anti-noise capability and reliability of MWD monitoring in cold-region geotechnical engineering.
- By delivering highly accurate, real-time stratigraphic profiles, this method establishes a crucial technical foundation for optimizing differentiated explosive charging, thereby reducing hazardous blasting effects and promoting energy-efficient, green mining operations.

**Abstract:**

To address the critical challenges of lithology acquisition and low blasting refinement under extreme low temperatures and varying thermal conditions in high-altitude environments, this study develops a real-time dynamic identification method for rock-like interfaces using Measurement While Drilling (MWD) technology. The scope of this research involves the use of a self-developed indoor digital drilling experimental platform to simulate both ambient and freezing (−20 °C) conditions. Procedures included conducting comprehensive comparative drilling experiments on various rock-like materials with distinct strength levels to evaluate their mechanical responses during penetration. The major findings reveal a significant influence of low-temperature hardening effects on MWD parameters; specifically, the frozen state notably increases drilling torque and feed pressure while simultaneously decreasing the stable rotational speed of the drill bit. To resolve the feature parameter drift induced by temperature variations, a novel interface recognition algorithm is proposed that integrates Z-score normalization, change-point detection, and multi-dimensional spatial clustering. Through a dual-detection mechanism involving both single-point and cumulative features, the algorithm effectively captures precise mutation information during rock layer transitions. It further incorporates multi-dimensional indicators, such as consistency, change intensity, and point density, to perform comprehensive weighted scoring. Experimental results demonstrate that the proposed algorithm effectively eliminates the systematic offset of parameters caused by temperature fluctuations. The prediction error at both “strong-weak” and “weak-strong” transition interfaces is maintained within 1.5 mm, which significantly improves the accuracy and robustness of interface recognition under complex and varying working conditions. These key conclusions provide essential technical support for the implementation of differentiated charging and green refined mining operations, ensuring greater energy efficiency and environmental protection in cold-region engineering.

## 1. Introduction

Accurate rock mass strength data is vital for optimizing fragmentation and reducing energy waste in open-pit blasting. In high-altitude regions, extreme cold and freeze–thaw cycles induce nonlinear hardening in rock mechanical properties, rendering traditional methods—such as borehole sampling and physical logging—inefficient and hazardous [1]. Consequently, obtaining deep stratigraphic information dynamically under varying temperatures is essential for refined, green mine construction.

Measurement While Drilling (MWD) technology enables in situ stratigraphic acquisition by monitoring mechanical parameters [2]. Based on Teale’s [3] Mechanical Specific Energy (MSE) theory, researchers have integrated machine learning [4,5,6,7,8] and neural networks to enhance lithology prediction and strength estimation. For instance, integrating Exploratory Data Analysis (EDA) with Random Forest models has significantly improved lithology classification accuracy [9], while advanced deep learning architectures like DBO-BiLSTM combined with wavelet denoising have achieved robust recognition rates for complex strata [10]. Notable advancements include KAN-optimized identification [11], vibration-signal CNN models [12], and multi-information fusion [13,14,15]. Recent fusion approaches have expanded beyond mechanical data to include seismic sensors (e.g., accelerometers and geophones) for identifying fine structural interfaces [16], and integrating Cone Penetration Test (CPT) probes into auger systems for real-time soil resistance monitoring [17]. Furthermore, fine detection methods analyzing torque frequency distribution [18] and numerical studies on drilling parameter dynamics under varying confining pressures [19] have deepened the quantitative understanding of rock-machine interactions [20]. Advanced algorithms—including random forests, XGBoost, and LightGBM—now support real-time geological characterization and engineering applications such as fragmentation prediction and grouting evaluation [21,22,23,24,25,26]. Such characterization is crucial for applications like slope stability analysis, where identifying microscopic stiffness mismatch and hydro-weakening directly informs macroscopic safety assessments [27]. Furthermore, the use of physics-based observers [28], multivariate analysis [29,30], and compound parameters [31] has improved system perception and modeling reproducibility [32,33,34,35,36,37]. Additionally, to ensure sensor reliability in extreme downhole environments, researchers have developed robust compensation methods, such as thermo-mechanical coupling inversion algorithms [38] and Modified Slime Mold Algorithm (MSMA) optimized piezoresistive compensation [39], successfully mitigating severe temperature-induced measurement drift. Furthermore, the potential of artificial intelligence in underground engineering extends far beyond drilling applications. Recent advancements have demonstrated that sophisticated machine learning architectures, particularly physics-informed neural networks (PINN) and generalization-oriented fusion models, possess exceptional capabilities in complex excavation scenarios. For instance, these advanced algorithms have been successfully deployed to optimize shield machine attitude planning, accurately estimate tunnel lining loads in elastic soils, and provide real-time predictions for both TBM operational loads and tunnel-induced surface settlements [40,41,42,43].

Despite these advancements, existing MWD research primarily focuses on ambient conditions or high-temperature deep wells, overlooking the dramatic physical changes rocks undergo in sub-zero temperatures [44,45]. Low-temperature hardening and ice–rock coupling exert complex interference on bit penetration, leading to significant parameter shifts and insufficient robustness in traditional models. Moreover, current normalization algorithms lack sensitivity during initial “collaring” stages and exhibit poor stability when processing noisy, long-sequence data in extreme environments. To address these gaps, this study utilized a self-developed digital drilling platform to compare rock responses under ambient and freezing conditions. We propose an interface recognition algorithm combining change-point detection and multi-dimensional spatial clustering. By employing Z-score normalization to eliminate temperature-induced feature drift and integrating multi-dimensional weighted scoring, this research provides a technical foundation for differentiated charging and refined green mining in high-altitude, cold-region environments.

## 2. Experiment and Method

### 2.1. Miniature Indoor Digital Drilling System

**Figure 1:**
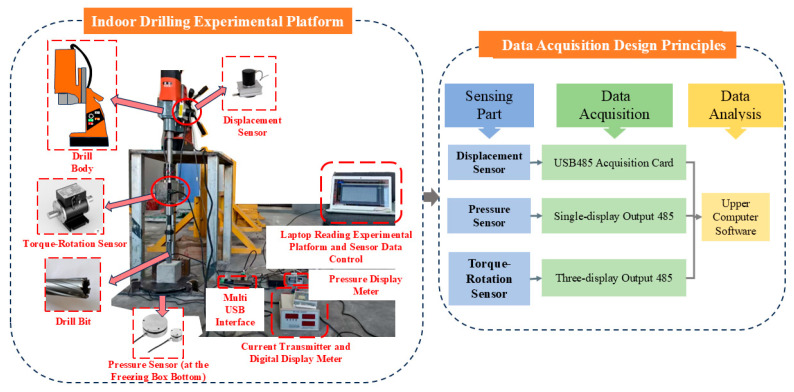
Indoor drilling experimental setup and data acquisition design principles.

Through a systematic review of the literature, it was known that drilling parameters such as torque, drilling pressure, rotational speed, and drilling speed during the drilling process were closely related to the mechanical properties of the rock. These parameters served as important indicators for evaluating rock mechanical properties and predicting blasting effects. Due to the difficulty of modifying drilling rigs on-site and the lack of a comprehensive MWD data acquisition system, related data collection was quite challenging. To accurately capture the dynamic mechanical responses of the rock mass during the drilling process, the self-developed digital drilling platform is equipped with a suite of high-precision sensors, as shown in Figure 1—including a displacement sensor, a torque and rotational speed transducer, and a pressure load cell, the detailed technical specifications of which are summarized in Table 1.

The experimental platform consisted mainly of the drilling system and the data monitoring system. By installing corresponding displacement sensors, pressure sensors, and torque-rotation sensors on the drill rig, the drilling parameters including drilling speed (V), drilling pressure (F), rotational speed (N), and torque (T) during the drilling of rock samples were precisely monitored. The displacement sensor was installed with one end on the drill body and the other end fixed on the rock sample holder, allowing for accurate detection of displacement-time curves during the drilling process. The torque-rotation sensor, through the modification of an adapter, was installed between the drill rods to collect torque and rotational speed in real-time during drilling.

Unlike traditional top-drive sensing layouts that are susceptible to mechanical transmission losses and rotational vibrations from the drill string, the pressure sensor in this study was strategically positioned directly beneath the sample chamber. According to Newton’s third law, this bottom-mounted load cell directly measures the vertical reaction force transmitted through the rock mass. This configuration effectively isolates the mechanical noise originating from the drill motor and accurately captures the true instantaneous penetration resistance exerted by the rock.

During drilling, the magnetic switch at the bottom of the drill rig was activated, allowing the drill rig to attach to the steel plate of the experimental platform to ensure stability during the drilling process. The drilling stroke was set to 170 mm.

### 2.2. Sample Mixture Ratio and Layered Sample Design

Due to the limited stroke of the drill rig and the thickness of the rock layers in the field blasting area, similar simulation experiments were used, where rock-like samples with similar strength were used to replace actual rock layers, achieving the goal of identifying layer interfaces.

Cubic molds with dimensions of 100 mm × 100 mm × 100 mm were selected to create four different strength single-homogeneous drilling samples and composite rock layer drilling samples. Using 32.5 composite Portland cement, sand and gravel (particle size < 5 mm), and water, rock-like blocks and corresponding samples with strengths of C5, C10, C15, and C20 were cast. The cement-sand ratios of the different strength samples are shown in Table 2.

To explore the dynamic response characteristics of MWD parameters at different temperatures, two temperatures were set. After 28 days of natural curing, four rock-like samples with different strengths were frozen at −20 °C in an automatic low-temperature freeze–thaw testing machine. These were then subjected to mechanical and digital drilling experiments, along with the four different strength samples at ambient temperature.

The composite rock-layer samples consisted of four groups: A+D+C and C+B+D under ambient temperature conditions, as well as A+D+C and C+B+D under −20 °C conditions. During the drilling process, the drill rig could pass through different strength differences and experienced strength variations of “weak-strong-weak” and “strong-weak-strong,” simulating the process of drilling through rock layer interfaces, thereby verifying the robustness of the recognition.

When making layered rock-like samples, it was crucial to ensure that the interfaces between layers were clear and tightly bonded, avoiding uneven strength distribution or unclear layering. First, the sample of the first strength grade was mixed evenly according to the ratio and poured into the mold with a thickness of 30 mm. It was poured in multiple stages, and a vibrator was used to remove air bubbles and ensure a smooth surface for the next layer. Before the first layer of cement mortar set, the second strength grade sample was poured into the mold, also to a height of 30 mm, and the vibration process was repeated. Then, before the second layer set, the third strength grade sample was poured into the mold, using the same method as the first two layers, with a height of 40 mm. The layered samples were placed indoors for 28 days of curing. After curing, some of the layered samples were placed in a freezing box set to −20 °C to simulate the environmental characteristics of rocks in high-altitude low-temperature conditions.

**Figure 2:**
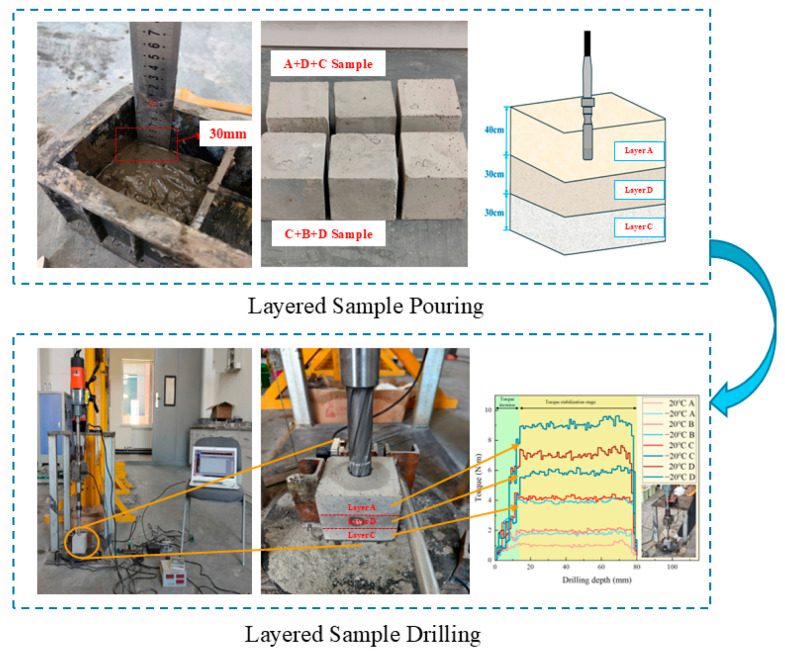
Preparation and drilling of layered samples.

As shown in Figure 2, based on the drilling verification experiments conducted on single-layer samples, layered sample drilling experiments were carried out. The initial rotational speed was set to 350 r/min, and other MWD parameters were monitored during the drilling process. To eliminate potential systematic errors caused by fluctuations in experimental equipment or operating conditions, four identical layered specimens were prepared for each combination, and only one hole was drilled in the center of each specimen to prevent micro-crack interference and boundary effects, thereby improving the reliability of the experimental results.

## 3. Experimental Results

### 3.1. Changes in Drilling Parameters of Samples with Different Mixture Ratios

**Figure 3:**
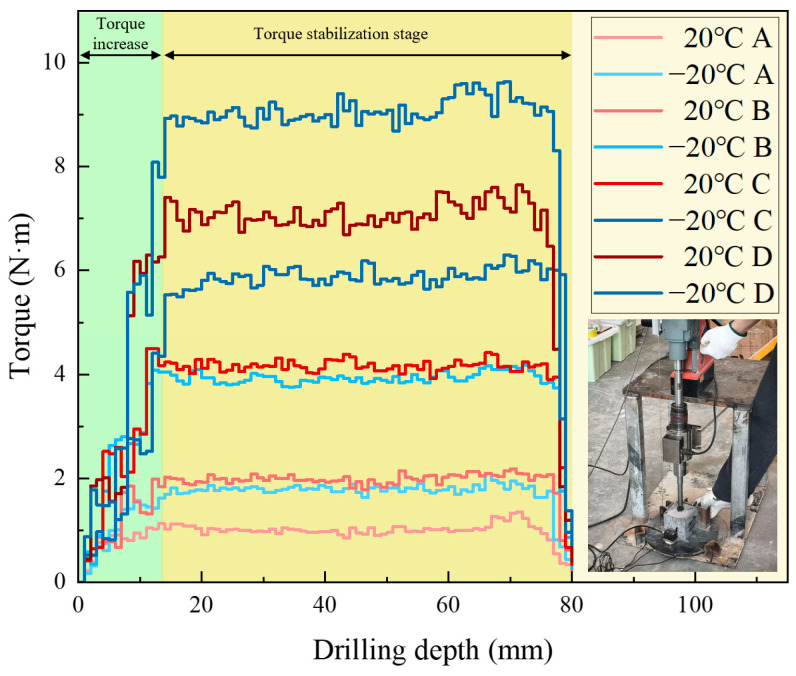
Drilling torque staircase curve for samples with different strengths under ambient and low-temperature conditions.

Figure 3 shows the torque variation trend required for drilling different strength samples under temperature variation conditions. From the torque-depth curve, it can be observed that the drilling torque initially increases sharply and then stabilizes. In the early stage of drilling, when the drill bit first contacts the sample surface, it must overcome significant cutting resistance, causing a sharp rise in torque. As drilling progresses and the contact area between the sample and the drill bit increases, frictional and cutting forces gradually stabilize, and the drilling torque tends to become steady. Since the torque data stabilizes after 10 mm of rock penetration, the drilling stroke is set to 80 mm to avoid the abnormal data interference caused by the drill bit penetrating the rock.

Comparing the drilling of 4 different strength rock samples, it was found that the lower the strength, the smaller the torque required for drilling, indicating better drillability of the low-strength samples. On the other hand, high-strength samples require greater torque to overcome resistance during drilling. Analysis of the low-temperature drilling torque shows that under ambient temperature conditions, the torque is relatively low, while at −20 °C freezing conditions, the torque increases for samples of different strengths compared to ambient temperature conditions. This indicates that low temperatures enhance the hardness of the samples, increasing the frictional and cutting resistance between the drill bit and the sample, and requiring more torque to complete the drilling.

**Figure 4:**
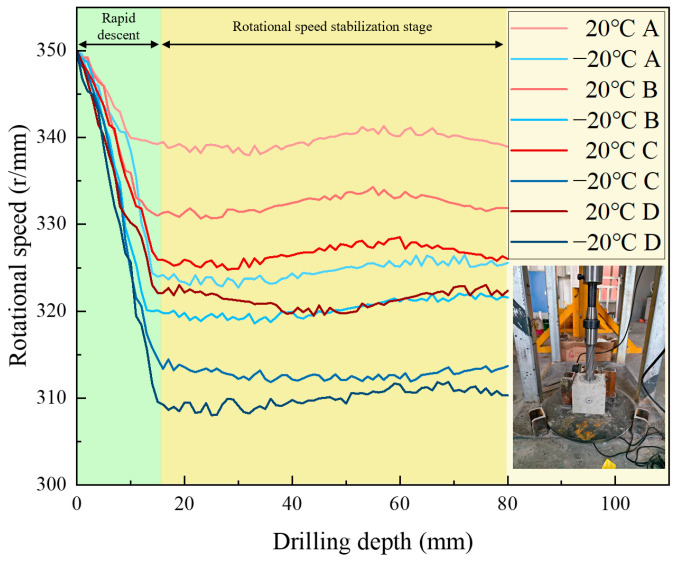
Drilling rotational speed curve for samples with different strengths under ambient and low-temperature conditions.

The drill rig was set to an initial rotational speed of 350 r/min. Real-time monitoring via the digital display showed that during the drilling of rock samples with different strengths, the rotational speed decreased to varying extents. Once it reached a certain value, the speed remained relatively stable or fluctuated within a small range. From the analysis of Figure 4, it can be observed that during the early stage of drilling (0–15 mm depth), the drill bit experiences significant frictional and cutting resistance when contacting the sample surface. In this stage, the drill bit must overcome the hardness of the sample’s surface and the reactive force during cutting, which leads to a sharp decrease in rotational speed. As drilling progresses and the drill bit gradually penetrates deeper into the rock, the cutting conditions stabilize, and the contact area between the sample and the drill bit, along with the frictional force, reaches a dynamic equilibrium, resulting in a gradual stabilization of the rotational speed. However, the stable value varies depending on the strength characteristics of the rock and whether it is frozen.

There are clear differences in the stable rotational speeds exhibited by rock samples with varying strengths during drilling. The samples with lower strength generally have higher stable rotational speeds compared to higher-strength rocks. This is because the internal structure of low-strength samples is relatively loose, with weaker resistance to cutting, allowing the drill bit to maintain a relatively higher speed. In contrast, high-strength samples have a denser internal structure and stronger resistance to cutting, leading to greater resistance against the drill bit during drilling, which results in lower stable rotational speeds. At ambient temperature, all rock samples exhibit relatively high stable rotational speeds. Under freezing conditions, as the temperature decreases, the stable rotational speeds gradually decrease, with a more significant reduction. This is due to the low-temperature freezing, which causes the internal moisture of the samples to freeze and expand. The water in the pores turns into ice, increasing the sample’s density and rigidity. This hardening effect makes the sample more difficult to cut at low temperatures, resulting in a decrease in stable rotational speed.

**Figure 5:**
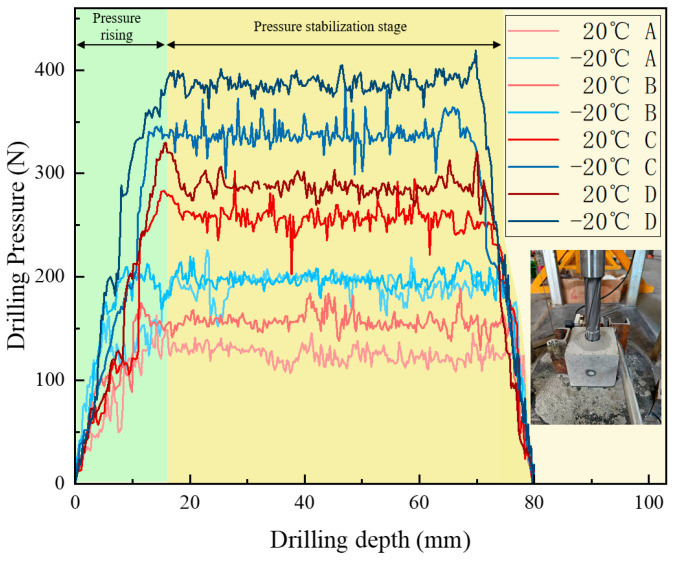
Drilling pressure curve for samples with different strengths under ambient and low-temperature conditions.

As shown in Figure 5, the trend of drilling pressure required for different strength samples under various temperature conditions is displayed. From the figure, it can be observed that the pressure variation trend is similar to that of the drilling torque, showing an initial sharp increase followed by relative stabilization. This reflects the change in frictional forces during the initial contact and cutting process between the drill bit and the sample. As drilling progresses, the contact area between the drill bit and the sample increases, and the cutting resistance and friction gradually stabilize, leading to a smoother drilling process. For rock samples of different strengths, low-strength samples require less pressure under both ambient and low-temperature conditions. As the strength of the rock increases, the resistance to the drill bit increases, requiring more drilling pressure to proceed smoothly. Under −20 °C freezing conditions, the overall drilling pressure shows an upward trend, indicating that the reduction in temperature significantly enhances the strength of the sample. The frictional and cutting resistance between the drill bit and the sample increases, requiring higher pressure for effective drilling.

### 3.2. Low-Temperature Hardening Effect of Samples

**Figure 6:**
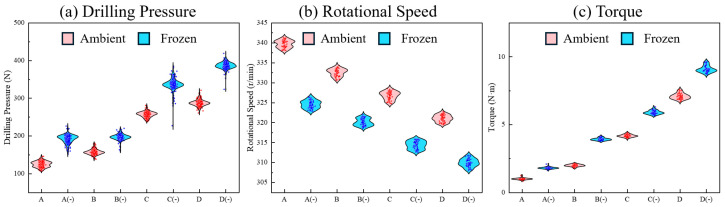
Distribution characteristics of drilling process parameters under ambient and frozen conditions: (**a**) drilling pressure; (**b**) rotational speed; and (**c**) torque.

Temperature variation typically has a significant impact on the mechanical properties of the samples, which in turn affects the stability of key parameters during the drilling process. By comparing the drilling data of different samples under ambient and −20 °C freezing conditions, the influence of extreme temperatures on signal volatility can be quantitatively evaluated. Instead of relying solely on mean baseline shifts, analyzing the Interquartile Range (IQR) data (Table 3) alongside the distribution profiles in the violin plots (Figure 6) provides a precise measure of mechanical fluctuation.

The data demonstrates that low temperatures distinctly enhance the hardness and brittleness of the samples. This ice–rock coupling effect not only increases the absolute cutting resistance but drastically amplifies the high-frequency fluctuations experienced by the drill bit. For instance, the torque IQR for the high-strength sample (Type D) expands significantly from 0.2712 at ambient temperature to 0.3202 under freezing conditions. Similarly, the drilling pressure IQR for sample D experiences a notable surge from 8.00395 to 9.50776. Even for medium-strength specimens like sample C, the torque and pressure IQRs widen from 0.1317 to 0.1691 and 9.17309 to 9.71533, respectively. These widened IQR values visually correspond to the vertically stretched and elongated profiles of the frozen samples in the paired violin plots. This structural widening indicates that the drill bit encounters highly erratic cutting resistance and intense, localized mechanical shocks in sub-zero environments. Although only a single drilling test was presented for each homogeneous specimen in the IQR analysis, the continuous MWD time-series data exhibited extreme stability over the entire 80 mm penetration depth. This longitudinal stability strongly validates the internal homogeneity of the prepared specimens and effectively rules out statistical contingency.

The rotational speed stability is correspondingly compromised by the low-temperature hardening effect. The rotational speed IQR for sample C increases from 1.5218 at ambient temperature to 1.877 under −20 °C conditions, while sample B’s IQR increases from 1.5534 to 1.7642. This amplified dispersion in rotational speed underscores that the freezing-induced hardening effect makes the sample significantly more difficult and unstable to cut.

Consequently, the severe signal volatility and single-point jumps induced by sub-zero conditions heavily interfere with traditional threshold-based data interpretation. This quantitatively justifies the necessity of deploying advanced noise-filtering and compensation algorithms to accurately extract true stratigraphic interfaces from highly fluctuant MWD sequences.

### 3.3. Layered Sample Drilling Experimental Results

By monitoring the rotational speed variation with drilling depth for two different strength combination layered samples under both ambient and freezing conditions (−20 °C), the dynamic response of the drilling process was analyzed.

**Figure 7:**
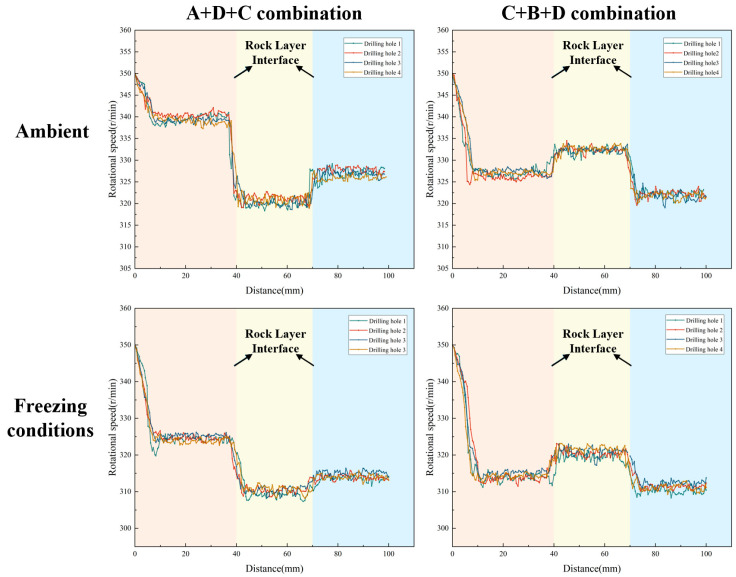
Rotational speed curve of layered rock samples under ambient and low-temperature conditions. The different background colors indicate the corresponding stratigraphic intervals along the drilling depth.

As illustrated in Figure 7, the rotational speed exhibits distinct, step-like responses corresponding to the pre-designed stratigraphic transitions. Under ambient conditions, the rotational speed adjusts dynamically to the varying cutting resistances of the rock layers. For instance, across both the A+D+C and C+B+D combinations, the speed drops abruptly when the drill bit enters a higher-strength layer (e.g., layer D) and recovers rapidly when transitioning into a weaker layer. Crucially, while the relative trend of these speed mutations remains consistent under −20 °C freezing conditions, the absolute baseline of the rotational speed is systematically lowered across all layers. This baseline shift physically demonstrates that the low-temperature environment significantly enhances the rock’s rigidity and cutting resistance. Nevertheless, the distinctive transient step-changes at the 40 mm and 70 mm interfaces are perfectly preserved, indicating that relative speed variations remain a reliable boundary indicator despite extreme thermal interference.

**Figure 8:**
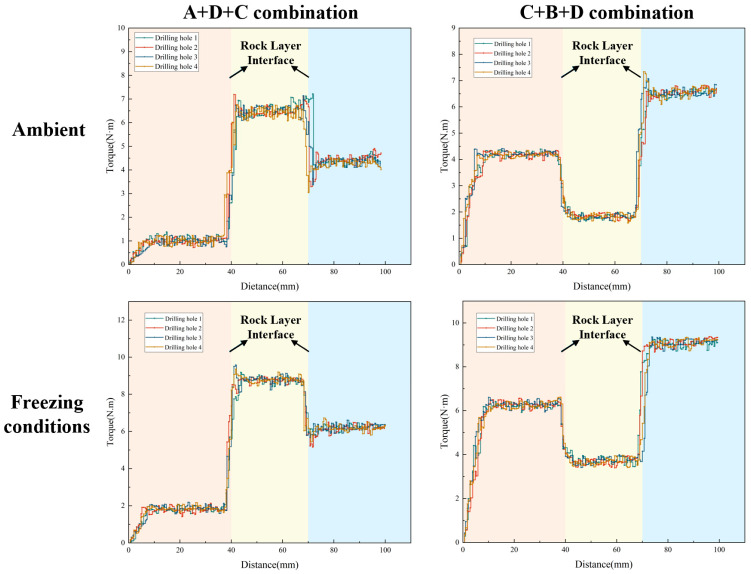
Torque staircase curve of layered rock samples under ambient and low-temperature conditions. The different background colors indicate the corresponding stratigraphic intervals along the drilling depth.

Similarly, the torque staircase curves (Figure 8) demonstrate highly sensitive, instantaneous mechanical responses to structural boundaries. Rather than exhibiting gradual drifts, the torque surges abruptly when the drill bit transitions from weak to hard strata—such as the nearly seven-fold increase (from approximately 0.94 N·m to 6.64 N·m) observed at the A-to-D interface under ambient conditions. Under freezing conditions, the ice–rock cementing effect induces a significant upward shift in the overall torque baseline. For example, the absolute torque required to penetrate layer D is notably elevated compared to its ambient counterpart. Despite this systematic temperature-induced baseline elevation, the relative magnitude of the transient mutations at the lithological boundaries remains stark and easily identifiable, laying a robust physical foundation for the subsequent feature extraction algorithm.

**Figure 9:**
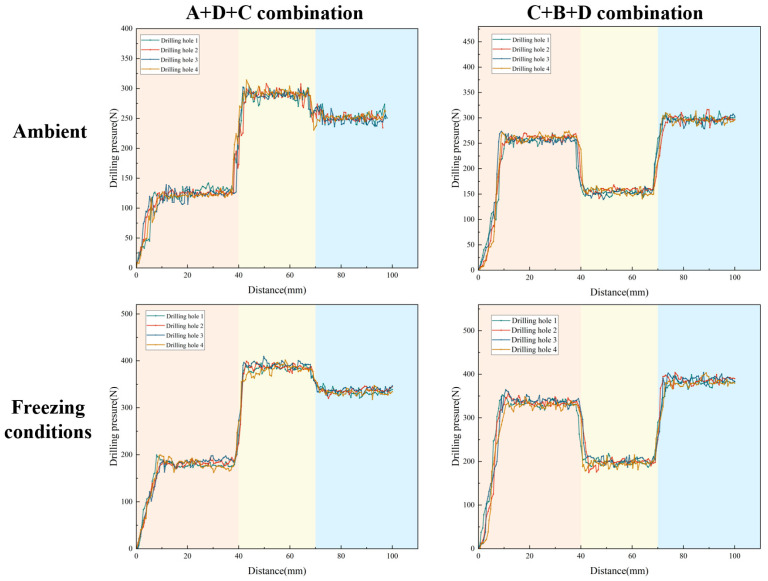
Drilling pressure curve of layered rock samples under ambient and low-temperature conditions. The different background colors indicate the corresponding stratigraphic intervals along the drilling depth.

The drilling pressure dynamics (Figure 9) further corroborate the sensitive mechanical response of the MWD system to deep stratigraphy. Consistent with the torque behavior, the feed pressure exhibits sharp, discrete jumps precisely at the 40 mm and 70 mm interfaces across both sample combinations. The transition into harder rock layers necessitates an immediate compensatory surge in vertical thrust to maintain penetration efficiency. When subjected to sub-zero conditions (−20 °C), the absolute drilling pressure required to penetrate each respective layer increases systematically, reflecting the enhanced macroscopic compressive strength of the frozen rock mass. Importantly, the abrupt pressure mutations precisely mirror the geological transitions without phase delays. This confirms that the relative step-changes in feed pressure are highly resilient to extreme thermal baseline shifts, serving as excellent primary data for the downstream change-point detection modules.

### 3.4. Rock-like Interface Recognition Algorithm Based on Mutation Detection and Clustering

**Figure 10:**
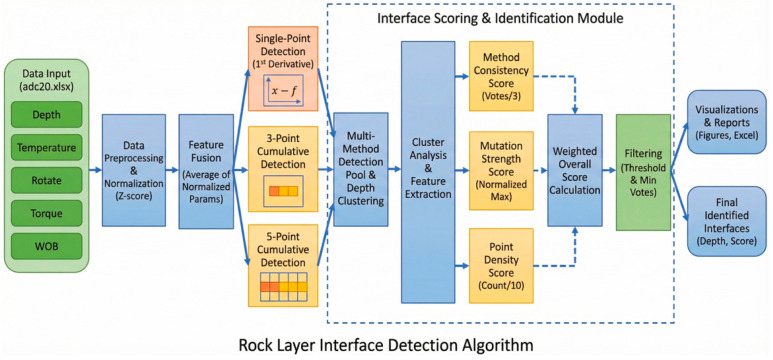
Flowchart of the rock-like interface recognition algorithm based on dual-mechanism detection and clustering.

As demonstrated by the significant IQR expansion in Section 3.2, the ice–rock coupling effect under sub-zero conditions induces severe high-frequency volatility. To overcome this challenge, a specialized multi-method fusion algorithm was developed. The algorithm comprises three core modules: temperature-drift compensation, dual-mechanism change-point detection, and multi-dimensional spatial clustering, as shown in Figure 10.

Given that freezing conditions induce a systematic base-value shift in mechanical parameters, directly applying threshold-based detection would lead to severe calibration errors. The algorithm first employs Z-score normalization on the multi-dimensional MWD time-series data. As shown in Equation (1):

(1) $$z_{i}=\frac{x_{i}-\mu }{\sigma }$$

*z_i_* is the Z-score normalized feature value of the corresponding sensor parameter at depth *i*; *x_i_* is the original absolute measurement value of the sensor at depth *i*; *μ* and *σ* are the global mean and standard deviation of the specific sensor’s time-series data across the entire drilling depth of the current borehole.

This step acts as a dynamic compensation mechanism, eliminating the absolute quantitative differences caused by temperature variations and dimensional mismatches across different sensors. The normalized signals are then fused into a composite feature sequence, ensuring that the subsequent detection focuses solely on relative mechanical mutations rather than thermal baseline drifts. As shown in Equation (2):

(2) $$F_{i}=\frac{1}{m}\sum_{j=1}^{m}z_{i}^{\left(j\right)}$$

*F_i_* is the composite feature value at depth *i*.

To address the highly volatile signal noise generated by brittle fracturing at low temperatures, a dual-mechanism detection approach combining single-point and cumulative analyses was implemented.

Single-Point Detection: This method captures instantaneous signal variations by calculating the local gradient, offering extreme sensitivity to sharp stratigraphic transitions. However, it is prone to triggering false positives when encountering transient drilling vibrations. As shown in Equation (3):

(3) $$\Delta F_{i}=F_{i}-F_{i-1}$$

$\Delta F_{i}$ is the single-point mutation gradient. It represents the transient variation in the composite feature value at depth *i* relative to the preceding depth point, utilized to capture highly abrupt micro-stratigraphic interfaces.

Cumulative Detection: To counteract the false positives, a cumulative change-point detection filter is applied. By continuously evaluating the accumulated deviation of the signal from its historical mean, this method smooths out localized transient spikes. It acts as a robust low-pass filter, effectively ignoring the single-point jumps caused by localized hard inclusions or ice lenses, and only responding to sustained macro-mechanical changes indicative of a true layer interface. As shown in Equation (4):

(4) $$\begin{array}{c} C_{i}=\frac{1}{w}\sum_{k=i}^{i+w-1}F_{k}-\frac{1}{w}\sum_{k=i-w}^{i-1}F_{k} \end{array}$$

*C_i_* is the cumulative feature deviation at depth *i*, w is the sliding window size for the cumulative calculation. Based on the data acquisition frequency and the penetration characteristics of the miniature drill rig, it is empirically set to 3 or 5 in this study.

The final module aggregates the candidate change-points identified by the dual-mechanism pool and performs depth-based spatial clustering. To definitively distinguish true geological interfaces from mechanical “drilling noise” (such as the severe instability commonly observed during the 0–15 mm initial collaring phase), the algorithm introduces a multi-dimensional weighted scoring system. Each potential interface is evaluated based on three indicators: method consistency score (*V_i_*), mutation strength score (*M_i_*), and spatial point density (*D_i_*). A strict filtering mechanism, utilizing minimum voting thresholds, ensures that isolated noise peaks are systematically rejected, retaining only the most statistically reliable rock-like interfaces. As shown in Equation (5):

(5) $$Score_{i}=W_{c}\cdot V_{i}+W_{m}\cdot M_{i}+W_{d}\cdot D_{i}$$

where *V_i_*, *M_i_*, and *D_i_* represent the normalized Method Consistency Score, Mutation Strength Score, and Point Density Score at depth i, respectively. The variables *W_c_*, *W_m_*, and *W_d_* are the corresponding weight coefficients. Based on the statistical distribution and prior empirical tuning from extensive laboratory drilling tests, the weight coefficients in this study were optimally set to *W_c_* = 0.4, *W_m_* = 0.4, and *W_d_* = 0.2. This configuration prioritizes the algorithmic consensus and the absolute mechanical mutation magnitude, while utilizing spatial point density as a secondary supplementary filter to effectively eliminate isolated transient mechanical noise.

### 3.5. Validation of Interface Recognition Performance Under Low-Temperature Hardening Conditions

To verify the stability of the algorithm under various temperature conditions, this section focused on the analysis of the identification accuracy and comprehensive scoring results for the two rock-like combinations, A+D+C and C+B+D. By comparing the feature value curves under ambient and freezing (−20 °C) conditions, the capability of the algorithm to suppress temperature-induced feature drift was further evaluated.

As shown in Table 4, during the drilling monitoring experiment of the rock-like combination A+D+C, the algorithm demonstrated good spatial localization accuracy and physical sensitivity. Under ambient temperature, a sharp positive characteristic change was captured near the A–D interface at approximately 40 mm, with the predicted location concentrated between 38.77 mm and 39.36 mm. The deviation from the designed interface was controlled within 1.5 mm. When the drill bit transitioned from the lower-strength layer A to the higher-strength layer D, the normalized values of torque, drilling pressure, and rotational speed increased sharply, and the recognition score reached 1.0.

It is worth noting that the initial 0–15 mm collaring data was not artificially excluded from the algorithm’s input. Instead, the multi-dimensional weighted scoring and minimum voting mechanisms automatically identified these highly erratic, unsustained mechanical fluctuations as ‘drilling noise’ rather than true stratigraphic interfaces, successfully filtering them out and further proving the model’s robustness.

Under −20 °C conditions, although the cementing effect of ice increased the overall rigidity of the layered rock-like samples, causing the base drilling torque to increase from an average of 7.03 N·m at ambient temperature to 9.00 N·m, the Z-score normalization effectively eliminated the influence of absolute value shifts. Experimental data show that the predicted interface position under low-temperature conditions was highly consistent with that under ambient temperature. Even at the 70 mm D–C interface, although the strength difference was slightly reduced compared to ambient temperature, the algorithm still managed to smooth fluctuations through the cumulative detection method, maintaining a high overall score above 0.783. This validates the robustness of the algorithm in extracting interface features under extreme temperature conditions.

During the drilling monitoring of the rock-like combination C+B+D (Table 5), at a drilling depth of approximately 40 mm, the drill bit experienced a sharp reduction in cutting resistance, and the algorithm identified a decrease in feature values. Under ambient temperature, the predicted average interface position was approximately 39 mm. Although signal fluctuations were amplified in the weaker rock-like layer, the algorithm effectively filtered out spurious feature points through depth clustering and the weighted scoring mechanism.

Notably, the signal interference during the early drilling phase (0–15 mm) was highlighted. In the experimental data from multiple drill holes, due to the impact and instability when the drill bit entered the sample, the algorithm identified a “pseudo-interface” at approximately 7 mm. However, the overall score of these interference points was relatively low, falling below the predicted rock-like interface score. This demonstrates the significant advantage of the multi-indicator scoring system in distinguishing “drilling noise” from “formation changes,” as it automatically removes high-frequency random fluctuations based on the minimum voting requirement.

## 4. Discussion

The results of this study demonstrate that sub-zero temperatures significantly enhance rock rigidity, leading to increased drilling torque and feed pressure, alongside decreased rotational speed. Unlike previous MWD studies that primarily focus on ambient conditions, our findings highlight the critical interference of ice–rock coupling on drill bit penetration. The proposed algorithm, utilizing Z-score normalization and change-point detection, successfully mitigates this temperature-induced parameter drift, maintaining interface prediction errors within 1.5 mm. This confirms our working hypothesis that multi-dimensional feature fusion and multi-dimensional spatial clustering can reliably isolate true geological transitions from temperature-induced anomalies.

In a broader context, accurate in situ stratigraphic acquisition is paramount for optimizing fragmentation and energy efficiency in extreme environments. The ability to dynamically characterize rock mass strength under freezing conditions provides a robust technical foundation for refined, green mine construction. By resolving the systematic offsets inherent in low-temperature MWD data, this approach enables the implementation of differentiated charging structures, ultimately reducing hazardous blasting effects, minimizing explosive waste, and protecting fragile cold-region ecosystems.

However, this study has limitations. The experiments utilized idealized rock-like samples and a miniature drill rig with a constrained stroke and a manual crank-feed mechanism. While this manual feed inherently introduces human-induced baseline wandering in absolute thrust compared to industrial constant-feed systems, it inadvertently validates the robustness of our proposed algorithm. From a signal processing perspective, manual feed inconsistency manifests as a low-frequency baseline wander, while sub-zero rock fracturing acts as high-frequency noise. The proposed Z-score normalization recenters the baseline, and the cumulative detection window acts as an effective band-pass filter, neutralizing both extreme high-frequency spikes and slow manual drifts, thereby solely responding to the true macro-mechanical step-changes (mutations) at rock interfaces. Additionally, the static freezing condition does not capture the dynamic freeze–thaw cycles present in high-altitude environments. Future research should prioritize field-scale validation using industrial-grade drilling equipment. Furthermore, subsequent studies must explore the influence of complex structural discontinuities—such as how ice-filled fractures affect the transmission of explosion energy. Building on this real-time geological data, integrating advanced machine learning models to conduct multi-dimensional evaluations of blasting effects in large-scale open-pit coal mines will be a crucial next step for the industry.

## 5. Conclusions

In this study, the MWD-based rock mass interface recognition method considering low-temperature hardening effects in cold regions was systematically investigated through indoor simulation experiments and algorithm development. The main conclusions are as follows:

(1) Experiments confirmed that the mechanical strength of the rock mass is significantly enhanced in a frozen state at −20 °C, leading to non-linear shifts in MWD dynamic responses: drilling torque and feed pressure increase substantially as temperature decreases, while the stable rotational speed exhibits a clear downward trend. This discovery provides a physical basis for correcting MWD recognition models in cold regions.
(2) To address the systematic parameter drift caused by low temperatures, a Z-score normalization algorithm was introduced to achieve dimensionless and centralized processing of multi-dimensional MWD parameters. Combined with change-point detection and multi-dimensional spatial clustering models, the algorithm effectively addresses the issues of low recognition accuracy and poor stability of traditional algorithms under temperature-varying environments, achieving accurate separation of “pseudo-interfaces” caused by changes in physical properties from actual geological interfaces.
(3) Experimental tests demonstrated that the algorithm is highly sensitive to rock interfaces of various strength combinations, with interface determination errors consistently less than 1.5 mm. In processing long-sequence drilling data, the algorithm exhibits excellent anti-noise capability and logical stability, meeting the requirements for high-precision dynamic identification in cold-region open-pit mines.
(4) The precise MWD-based interface recognition achieved in this study provides real-time rock occurrence information for high-altitude mines, supporting the optimized design of differentiated charging structures. This holds significant application value for enhancing energy utilization, controlling hazardous blasting effects, and protecting fragile ecosystems in cold regions.

## Figures and Tables

**Table 1:** Technical specifications of the sensors.

Structure	Indicator	Parameter
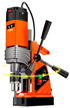	Drilling rig stroke	170 mm
	Rated voltage	220 V
	Rated power	1880 W
	Speed range	0–550 r/min
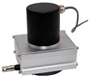	Range	0–1000 mm
	Accuracy	0.03 mm
	Overload capacity	120%
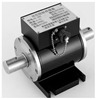	Torque range	0–100 N·m
	Rated voltage	24 V
	Speed range	0–500 r/min
	Accuracy	0.5%
	Overload capacity	150%
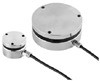	Pressure range	0–100 kg
	Accuracy	0.1%F.S
	Overload capacity	120%F.S

**Table 2:** Mixture ratios of samples with different strengths.

Specimen No.	Cement Content/%	Sand Content/%	Water Content/%
A	8.06	76.61	15.32
B	11.27	73.96	14.77
C	16	70.56	13.44
D	20.28	66.13	13.59

**Table 3:** IQR values of MWD parameters for different rock types under ambient and freezing conditions.

Type	Drilling Pressure	Rotational Speed	Torque
A	12.0294	1.3894	0.0597
A(-)	12.5077	1.5154	0.0621
B	7.41966	1.5534	0.1009
B(-)	7.67438	1.7642	0.1309
C	9.17309	1.5218	0.1317
C(-)	9.71533	1.877	0.1691
D	8.00395	1.4144	0.2712
D(-)	9.50776	1.6447	0.3202

**Table 4:** Change-point detection results and interface prediction results for A+D+C samples under ambient and low-temperature conditions.

Rock Layer	Hole No.	C1	C3	C5	Depth Range (mm)	Max Change Intensity	Predicted Position (mm)	Comprehensive Score	Feature Value Plot
A+D+C (Ambient)	1	11	10	13	35.28–42.02	0.6437	38.77	1	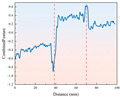
					63.26–72.02	0.5201	70.07	0.923	
	2	7	7	10	36.29–41.69	0.8885	38.93	1	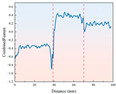
					70.00–70.34	0.3572	70.21	0.661	
	3	7	7	9	1.72–6.55	0.2374	4.3	0.48	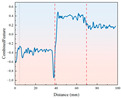
					36.29–41.01	0.7134	38.81	1	
					66.29–76.07	0.2455	69.66	0.784	
	4	13	13	13	36.29–41.69	0.4995	39.36	0.986	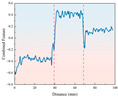
					64.94–70.34	0.5169	68.68	1	
A+D+C (−20 °C)	1	12	12	13	37.30–43.71	0.5187	39.78	1	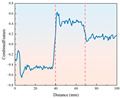
					67.98–69.66	0.1765	68.86	0.783	
	2	10	8	12	37.30–41.69	0.4371	39.17	1	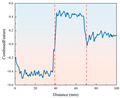
					67.98–77.75	0.2488	70.38	0.828	
	3	16	14	11	3.09–5.83	0.2047	4.94	0.47	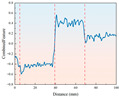
					37.30–42.02	0.573	39.45	1	
					67.98–73.71	0.2976	69.02	0.808	
	4	10	12	12	5.14–5.83	0.2186	5.3	0.598	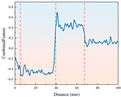
					37.30–42.02	0.6326	39.41	1	
					66.29–67.98	0.2869	67.36	0.701	

**Table 5:** Change-point detection results and interface prediction results for C+B+D samples under ambient and low-temperature conditions.

Rock Layer	Hole No.	C1	C3	C5	Depth Range (mm)	Max Change Intensity	Predicted Position (mm)	Comprehensive Score	Feature Value Plot
C+B+D (Ambient)	1	18	15	16	3.10–10.67	0.343	7.97	0.455	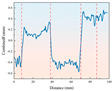
					32.92–39.33	0.539	38.33	1	
					65.96–72.02	0.533	70.23	0.996	
					84.16–93.93	0.240	86.9	0.505	
	2	12	14	16	5.17–9.31	0.550	7.03	0.626	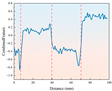
					38.99–43.71	0.443	39.64	0.802	
					68.65–71.69	0.676	70.53	1	
	3	14	18	17	1.72–5.86	0.328	7.54	0.553	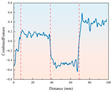
					38.31–39.33	0.427	38.77	0.889	
					66.29–72.70	0.519	69.07	1	
	4	11	11	12	6.90–6.90	0.2891	6.9	0.479	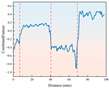
					38.99–46.74	0.5088	40.48	0.863	
					63.93–71.01	0.6723	68.99	1	
C+B+D (−20 °C)	1	14	11	12	1.03–6.90	0.4323	6.59	0.535	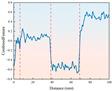
					37.98–41.01	0.4613	38.79	0.851	
					67.98–71.69	0.7351	68.99	1	
	2	17	13	13	5.17–15.73	0.3104	6.89	0.472	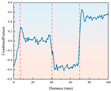
					34.94–43.03	0.3163	40.53	0.795	
					68.31–71.01	0.648	69.74	1	
	3	15	16	16	2.07–9.31	0.4322	5.98	0.86	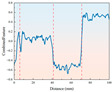
					40.00–47.75	0.4194	41.34	0.812	
					70.00–72.02	0.6657	71.07	1	
	4	18	16	15	4.14–10.00	0.2798	7.03	0.591	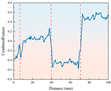
					37.30–41.01	0.4993	39.75	0.976	
					70.07–72.70	0.5305	70.67	1	

## Data Availability

Some or all data, models, or code that support the findings of this study are available from the corresponding author upon reasonable request.
